# Causative drugs for drug-induced cutaneous reactions in central China: a 608-case analysis^☆^^☆☆^

**DOI:** 10.1016/j.abd.2019.01.007

**Published:** 2019-10-26

**Authors:** Jun Zhao, Lei Hu, Lihua Zhang, Maosong Zhou, Lichen Gao, Lin Cheng

**Affiliations:** a School of Ophthalmology & Optometry Affiliated to Shenzhen University, Shenzhen, China; b Shenzhen Eye Hospital Affiliated to Jinan University, Shenzhen Eye Institute, Shenzhen, China; c Department of Pharmacy, Peking University People's Hospital, Beijing, China; d Department of Clinical Pharmacology, Xiangya Hospital, Central South University, Changsha, China; e Institute of Clinical Pharmacology, Hunan Key Laboratory of Pharmacogenetics, Central South University, Changsha, China; f Department of Dermatology, Changsha Eighth People′s Hospital, Changsha, China; g Department of Pharmacy, Department of Oncology, Cancer Institute, Changsha Central Hospital, Changsha, China; h State Key Laboratory of Ophthalmology, Zhongshan Ophthalmic Center, Sun Yat-sen University, Guangzhou, China; i Shenzhen Eyeis Visual Science Research Institute, Shenzhen, China; j School of Ophthalmology & Optometry, Eye Hospital, Wenzhou Medical University, Wenzhou, China

**Keywords:** Drug eruptions, Drug hypersensitivity, Drug-induced cutaneous adverse reactions, Drug-induced severe cutaneous adverse reactions, Pharmacovigilance

## Abstract

**Background:**

Reports regarding the causative drugs of drug-induced cutaneous adverse reactions in China are indistinct, such that different regions have reported the spectrum of drugs differs substantially in different clinical conditions.

**Objective:**

To explore the causative drugs that led to cutaneous reactions.

**Methods:**

Adverse drug reaction reports from central China were collected and divided into cutaneous adverse reactions and severe cutaneous adverse reactions groups. Cases were reviewed retrospectively for causative drugs.

**Results:**

The male:female ratio was equal in both cutaneous adverse reactions and severe cutaneous adverse reactions. In cutaneous adverse reactions (*n* = 482), the highest incidence happened between 51 and 60 years of age and the top three causative drugs were antibiotics (48%), Chinese medicine (16%), and allopurinol (9%). In severe cutaneous adverse reactions (*n* = 126), the highest incidence happened between 41 and 50 years of age and the top three causative drugs were sedative-hypnotics and antiepileptics (39%), antibiotics (22%), and allopurinol (15%). Carbamazepine was the most frequently used single-drug (16/18) in sedative-hypnotics and antiepileptics. β-lactams were the most frequently used antibiotics that induced both cutaneous adverse reactions and severe cutaneous adverse reactions.

**Study limitations:**

The small sample size, retrospective design, collection of cutaneous adverse reactions and severe cutaneous adverse reactions at different time frames and locations, and exclusion of patients taking more than five medications are limitations of the study.

**Conclusions:**

Gender does not affect cutaneous adverse reactions and severe cutaneous adverse reactions. The top three drugs to induce cutaneous adverse reactions are antibiotics, Chinese medicine, and allopurinol, while those that triggered severe cutaneous adverse reactions are sedative-hypnotics and antiepileptics, antibiotics, and allopurinol. Carbamazepine is the most frequent single drug that induces severe cutaneous adverse reactions. β-lactams are the most frequently used antibiotics that induce both cutaneous adverse reactions and severe cutaneous adverse reactions.

## Introduction

Drug eruption is a symmetric cutaneous reaction that may occur when patients are taking medicines. It is known as drug-induced cutaneous adverse reactions (CARs), drug dermatitis, or dermatitis medicamentosa. Drug eruption is usually mild and may disappear when offending drugs are withdrawn; however, some severe cases may be fatal. According to reports, drug eruption affects more than 7% of the general population and an estimated 7000 deaths occur.^1^, ^2^ The incidence of severe cutaneous adverse reactions (SCARs) is generally very low. Taking Stevens-Johnsons syndrome (SJS) and toxic epidermal necrolysis (TEN) for example, around 5.76 cases occur out of one million people per year.^3^, ^4^, ^5^ Once such reactions occur, the total mortality could be as high as 30%,^6^ 38%,^7^ or 32%,^8^ as stated by different reports.

Drugs known to induce severe drug eruption are mainly antibiotics, antiepileptic drugs, non-steroidal anti-inflammatory drugs (NSAIDs), and allopurinol. Data showed the main causative drugs were antibiotics (TEN, 40%; SJS, 40%) and pain relievers (TEN, 23%; SJS, 33%), and among antibiotics, sulfa and β-lactams were the most common causes of CARs.^9^, ^10^ The spectrum of drugs that induce CARs varied substantially in different clinical conditions. Identifying the causative drugs not only allows early cessation, but also improves the prognosis. Since the epidemiological data for CARs are limited in China at present, the authors conducted an analysis in hospitalized patients with drug eruption in central China to explore the leading drugs that cause CARs in Chinese population.

## Methods

### Patients

The study is a retrospective observational study. Four hundred and eighty two CARs patients admitted to Wuhan No. 1 Hospital from 2010 and 2011 and 126 SCARs patients admitted to Xiangya Hospital from 2009 to 2014 were assessed (Table 1). The patients’ general information, causative drugs, clinical manifestations, and outcomes were collected and analyzed. The diagnosis of drug eruption was made according to the patient's history of medication, clinical manifestations, and latent period by ruling out similar infectious skin diseases and rashes. Eruptions were diminished or relieved upon withdrawal of the sensitizing drug. The eruption manifestation was a typical symmetrical drug eruption, with bright red color and itch. If patients were taking multiple medicines, determining the sensitizing drugs may have been difficult. In this case, the suspected drug was determined by the doctors-in-charge. Only cases showing the definite causative drugs and taking no more than five total drugs were included. In CARs with probable or unknown drugs, the cases were excluded.

**Table 1:** Patient information from two different hospitals.

Hospital	Time (years)	Cases	Patient type
Wuhan No. 1 Hospital	2010–2011	482	Patients with drug eruption^a^, ^b^
Xiangya Hospital	2009–2014	126	Patients with severe drug eruption^a^, ^b^

a Unknown drugs that induce drug eruption were ruled out.

b Patients intake ≤ 5 total drugs were analyzed.

### Clinical manifestation classification

**Fig. 1:**
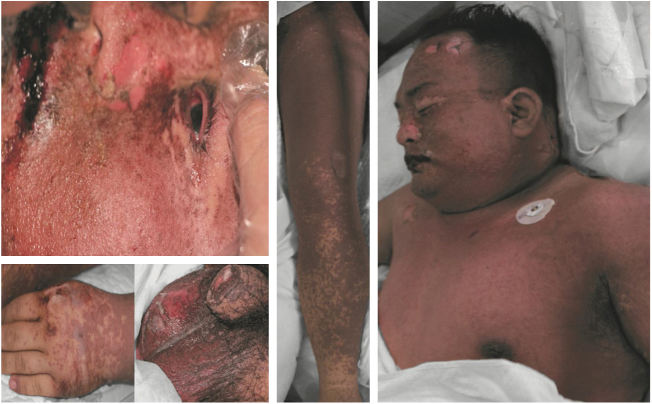
Dermatological manifestations of a patient with toxic epidermal necrolysis.

This study divided drug eruptions into two categories: (1) CARs: fixed drug eruption, urticarial drug eruption, measle-like or scarlatina-like drug eruption, eczematous drug eruption, purpura-like drug eruption, erythema multiforme, acneiform drug eruption, photosensitive drug eruption, etc. (2) SCARs: erythema multiforme major or SJS, drug rash with eosinophilia and systemic symptoms (DRESS), TEN, erythroderma, and exanthematous pustulosis.^9^, ^11^ One patient who presented TEN is shown in Fig. 1.

### Drug classification

The most common drugs that induce drug eruption were divided into the following categories: (1) antibiotics; (2) analgesic-antipyretics, such as aspirin, acetaminophen, etc.; (3) sedative hypnotics and antiepileptic drugs, such as phenobarbitone, phenytoin sodium, carbamazepine, etc.; (4) serum preparations and vaccines, such as tetanus antitoxin, rabies vaccine, snake antivenoms, etc.; (5) biological agents. In addition, the anti-hyperuricemia drug allopurinol, antithyroids, and phenothiazines were also included.

### Statistical analysis

All the numerical variables in this article were presented as mean ± standard error of the mean (SEM). Student's *t*-test was used on numerical variables of independent samples. The chi-squared test was used on categorical variables, *i.e*., enumerated group data. All data were analyzed by the SPSS v. 13.0 software package (SPSS Inc. – Chicago, IL, United States). A *p*-value < 0.05 was considered statistically significant.

## Results

### 482 cases of CARs

**Fig. 2:**
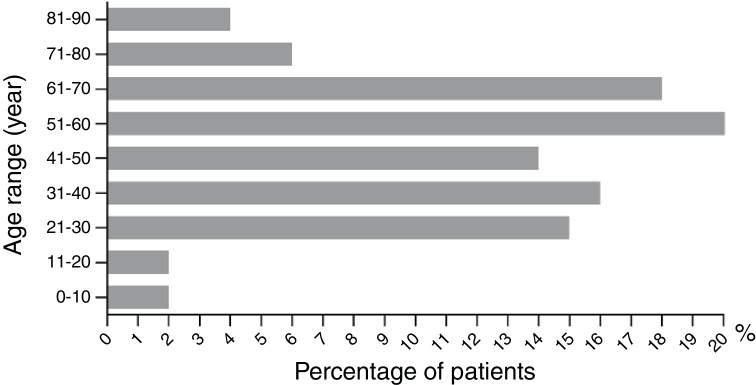
Age distribution of cutaneous adverse reactions (CARs). The chart illustrates the patient distribution at different age ranges (years old).

Among the 482 CARs cases in Wuhan No. 1 Hospital, the cases of male and female patients were equal, with no statistical difference (49%, 238/482 *vs.* 51%, 244/482) (*p* > 0.05). The age distribution between male and female were not statistically different (48.71 ± 1.90 *vs.* 46.24 ± 1.26 years old) (*p* > 0.05). The highest CARs incidence occurred between 51 and 60 years old, and the lowest happened between 71 and 80, 81 and 90, 0 and 10, and 11 and 20 years old (Fig. 2). Patients were divided into the single-medicine use group and the combined-medicine use group according to their causative drugs. The causative drugs were then subdivided into subcategories (Table 2). In the single-medicine use group, the most frequent drugs were antibiotics (48%, 201/420), followed by Chinese medicine (16%, 66/420) and allopurinol (9%, 38/420). These were followed by NSAIDs, sedative hypnotic antiepileptic drugs, nitroimidazole, antihypertensives and lipid-modifying agents, serums and vaccines, digestive system drugs, biological agents, antifungals, antithyroid preparations, and antiviral drugs. Other agents included anti-prostatic hyperplasia drugs, pesticides, mango, etc. In the combined-medicine use group, the most common combinations were antibiotics combined with other drugs. Antibiotics combined with antibiotics accounted for 34% (21/62). Antibiotics combined with other drugs, such as serum preparations, antipyretic analgesics, anticoagulants, antiviral drugs, and Chinese medicine accounted for 27% (17/62). The third place was antibiotics combined with nitroimidazole, accounting for 13% (8/62). Subsequently, it was Chinese medicine combined with another Chinese medicine, antiepileptic medicine combined with another antiepileptic medicine, and nitroimidazole combined with another nitroimidazole.

**Table 2:** The categories of causative drugs in 482 cases of cutaneous adverse reactions.

Group	Ranking	Causative drugs	ATC codes	Cases	% *vs.* subtotal	% *vs.* total
Single-medicine use	1	Antibiotics	A07AA, C05AB, D01AA, D06A, D06AX, G01AA, J02AA, J04AB, R02AB, R02AB	201	48%	
	2	Herbs and Chinese medicine	–	66	16%	
	3	Allopurinol	M04AA01	38	9%	
	4	NSAIDs	M01A, M01AX, S01BC	30	7%	
	5	Sedative-hypnotics and antiepileptics	N05C and N03	20	5%	
	6	Nitroimidazole	P01AB, P01CA	12	3%	
	7	Antihypertensives and lipid modifying agents	C02 and C10	12	3%	
	8	Serum and vaccine	G03GA03, J06AA03, J06AA06 and J07	9	2%	
	9	Digestive system drugs	A09	5	1%	
	10	Biological agents	B05A, L03AB, L03AC, L04AB, etc.	3	1%	
	11	Antifungals	D01	3	1%	
	12	Antithyroid preparations	H03B	3	1%	
	13	Antivirals	S01AD	1	0%	
	14	Others^a^	–	17	4%	
		Subtotal		420		87%
Combined-medicine use	1	Antibiotics + antibiotics	S01AA30	21	34%	
	2	Antibiotics + others	S01AA20	17	27%	
	3	Antibiotics + nitroimidazole	–	8	13%	
	4	Herb + herb	–	3	5%	
	5	Antiepileptics + antiepileptics	–	2	3%	
	6	Nitroimidazole + nitroimidazole	–	1	2%	
	7	Others^b^	–	10	16%	
		Subtotal		62		13%
Total				482		

The Anatomical Therapeutic Chemical (ATC) coding system divides drugs into different groups according to the organ or system on which they act, or their therapeutic and chemical characteristics.

a Anti-prostatic hyperplasia, pesticides, mango, etc.

b Serum products, non-steroidal anti-inflammatory drugs (NSAIDs), anti-tumor medicine, herbs, etc.

Among those antibiotics (Table 3), β-lactams were the most frequently used (46%, 92/201), followed by furan (22%, 44/201), lincomycin (12%, 24/201), quinolone (12%, 24/201), macrolides (4%), and sulfonamides (3%). In β-lactams, amoxicillin was the most frequently used (43%, 40/92). In furan antibiotics, the most common was nifuratel (95%, 42/44).

**Table 3:** The categories of causative antibiotics in 482 cases of cutaneous adverse reactions.

Drugs	ATC codes	Cases	%
β-lactams	J01C, J01CR, J01D	92	46%
Furan	D08AF, J01XE, P01CC	44	22%
Lincomycin	J01FF02	24	12%
Quinolone	J01M, J01MA, J01MB, S01AE	24	12%
Macrolides	J01FA	8	4%
Sulfonamides	A07AB, C03BA, C03CA, D06BA, G01AE, S01AB	7	3%
Others	–	2	1%
Total		201	100%

### 126 cases of SCARs

Among the 126 SCARs cases in Xiangya Hospital, we found 43.50% (385/885) of patients were taking more than five drugs. To this end, patients taking more than five drugs were excluded due to it being overly complicated to determine the culprit drugs and/or to perform statistical analysis.

**Fig. 3:**
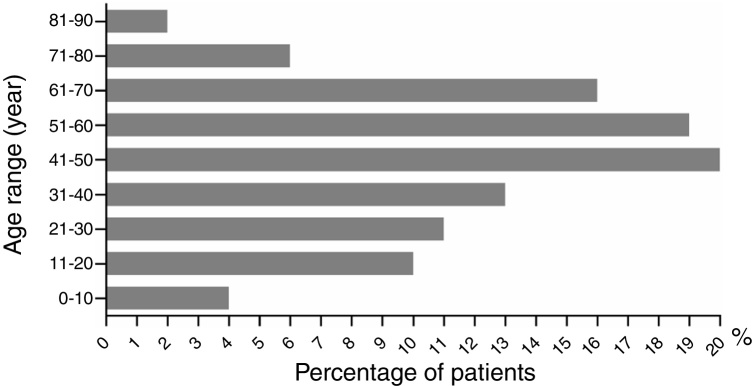
Age distribution of severe cutaneous adverse reactions (SCARs). The chart illustrates the patient distribution at different age ranges (years old).

The ratio of males to females was 1 (43%, 54/126) to 1.33 (57%, 72/126) (*p* > 0.05). Male age to female age was 48.70 ± 2.65 *vs.* 43.79 ± 2.30 years old (*p* > 0.05). Age ranged from 2 to 86 years old. Patients aged under 10 and those between 71 and 90 had the lowest incidence rates, with five (4%, 5/126) and ten cases (8%, 10/126), respectively (Fig. 3). The highest incidence occurred at the age between 41 and 50 years old, with a proportion of 16% (20/126). The dermatological types of SCARs are listed in Table 4. Severe erythema multiforme was the most common type of SCARs, accounting for 38% (48/126). The second place was exanthematous pustulosis, accounting for 26% (33/126). TEN was rare, accounting for 2% (3/126).

**Table 4:** The dermatological types of 126 cases of severe cutaneous adverse reactions.

SCARs types	Male	Female	Total	%
Severe erythema multiforme	23	25	48	38%
Exanthematous pustulosis	14	19	33	26%
Stevens-Johnsons syndrome	0	4	4	3%
Toxic epidermal necrolysis	2	1	3	2%
Erythroderma	2	0	2	2%
Another severe drug eruption	13	23	36	29%
Total	54	72	126	100%

The most common drugs in single-medicine use were sedative-hypnotics and antiepileptics, accounting for 39% (18/46) (Table 5). Antibiotics (22%, 10/46) and allopurinol (15%, 7/46) were the second and third categories that induce SCARs. They were followed by Chinese patent medicine or combined Chinese and drug medicine, nitroimidazole, dapsone, serum and vaccine, and digestive system medicines. Other agents such as fruits, vegetables, and diet pills were not included in the single-drug group, since they usually did not induce SCARs. Among sedative-hypnotics and antiepileptics, carbamazepine was the most frequently used single medicine (89%, 16/18). β-lactams were the most frequent antibiotics that induce SCARs.

**Table 5:** The categories of causative drugs in 126 cases of severe cutaneous adverse reactions.

Groups	Ranking	Causative drugs	ATC codes	Cases	% *vs.* subtotal	% *vs.* total
Single-medicine use	1	Sedative-hypnotics and antiepileptics	N05C and N03	18	39%	
	2	Antibiotics	A07AA, C05AB, D01AA, D06A, D06AX, G01AA, J02AA, J04AB, R02AB, R02AB	10	22%	
	3	Allopurinol	M04AA01	7	15%	
	4	Herbs and Chinese medicine	–	5	11%	
	5	Nitroimidazole	P01AB, P01CA	2	4%	
	6	Dapsone	J04BA02, D10AX05	2	4%	
	7	Serum and vaccine	G03GA03, J06AA03, J06AA06 and J07	1	2%	
	8	Digestive system medicines	A09	1	2%	
		Subtotal		46		37%
Combined- medicine use	1	Two drug combination	–	34	43%	
	2	Three drug combination	–	23	29%	
	3	Four drug combination	–	14	18%	
	4	Five drug combination	–	9	11%	
		Subtotal		80		63%
Total				126		

In combined-medicine use, since drugs were randomly combined, we only analyzed the frequency of duplex, triple, quadruple, and quintuple drug combinations (Table 5). Duplex drug combination accounted for 43% (34/80), triple drugs accounted for 29% (23/80), quadruple drugs accounted for 18% (14/80), and quintuple drugs accounted for 11% (9/80). The medicine combination of SCARs was more complicated than that of CARs, *e.g.*, some patients had antibiotics combined with antiviral drugs, serum preparations, and Chinese medicine.

## Discussion

In this study, we analyzed 608 cases of drug eruption in Chinese population from central China. 482 cases of CARs suggested that men and women had equal chance to develop CARs, and those with age between 51 and 60 had the highest incidence of CARs. The suspected drugs were mainly in the following ten categories: (1) antibiotics: β-lactams were the most frequently used antibiotics; (2) Chinese medicine or Chinese patent medicine, or combined Chinese and drug medicine; (3) allopurinol; (4) NSAIDs; (5) sedative hypnotic antiepileptic drugs; (6) nitroimidazole; (7) antihypertensives and lipid-modifying agents; (8) serum and vaccine; (9) digestive system drugs; (10) biological agents. This study showed the top three CARs-inducing medicine categories were antibiotics, Chinese medicine, and allopurinol. In antibiotics, β-lactams, furan, and clindamycin were the top three antibiotic subgroups that induce CARs. Consistent with previous reports, β-lactams were the most common antibiotics to induce drug eruption.^12^, ^13^, ^14^, ^15^ Widely used in China, Chinese medicine, including herbs, Chinese patent medicine, combined Chinese and drug medicine, and Chinese medicine injections ranked in the second place to induce CARs in central China. Due to improper use of Chinese medicine, pharmaceutical problems, specific ingredients, complex components of Chinese medicine, or perhaps interaction with drugs, drug eruption induced by Chinese medicine was commonly seen. If the ingredients of Chinese medicine are not clear, use of Chinese medicine combined with other medicine should be more careful, especially when patients have allergy history and multiple drug usage. Furan was not a common cause of drug eruption and ranked the second place in antibiotics to induce CARs. This may due to the nifuratel vaginal suppository, which was extensively used in Wuhan city. It suggests that Wuhan city should pay a special attention to the adverse reactions of β-lactams such as amoxicillin and the gynecological suppository medicine nifuratel due to their high adverse reactions, and consider changing to another vendor.

There were 126 cases of SCARs, which showed that SCARs were most common between 40 and 70 years old of age. Women had more cases of SCARs than men, but without statistical significance. The top three causative drugs to induce SCARs were sedative-hypnotics and antiepileptics, antibiotics, and allopurinol. Among sedative-hypnotics and antiepileptics, carbamazepine was the most frequently used drug, making it the most frequent single-drug that induce SCARs. β-lactams were the most frequently used antibiotic that induce SCARs. Allopurinol ranked number three to induce SCARs, which is consistent with previous reports that allopurinol is a common drug to induce SCARs.^16^, ^17^, ^18^, ^19^

Meanwhile, it was found that most SCARs patients used a multiple-drug combination and had a complex medication history. They had triple, quadruple, and quintuple drug use. Compared with the combined-medicine use group in CARs, SCARs patients had more drug combinations. Despite primary diseases, SCARs patients usually had more drug intake, which greatly enhanced the incidence of SCARs. Avoiding interaction among different medications is important to lower the incidence of SCARs.

Despite the findings above, this study has limitations. The small sample size is one of them. This study only analyzed the patients who took no more than five drugs in SCARs, since more than five drug intake cases were too sophisticated for either determining the culprit drugs or doing statistical analysis. In this way, some meaningful data were eliminated for these severe cases. This estimation may underrepresent SCARs in Xiangya Hospital.

## Conclusions

In central China, gender does not affect CARs and SCARs. The highest incidence of CARs occurs between 51 and 60 years old of age. The top three drugs to induce CARs are antibiotics, Chinese medicine, and allopurinol. Of antibiotics, β-lactams, nitrofurans, and lincomycin are the top three categories to induce CARs. However, SCARs are commonly seen between 41 and 50 years old of age. The top three drugs to induce SCARs in single-medicine use are sedative-hypnotics and antiepileptics, antibiotics, and allopurinol. Of the sedative-hypnotics and antiepileptics, carbamazepine is the most frequently used drug, making carbamazepine the most frequent single medicine to induce SCARs. β-lactams are the most frequently used antibiotics to induce SCARs. Despite these findings, this study has limitations. The small sample size, retrospective design, collection of the non-severe and severe CARs at different time frames and locations, and exclusion of patients taking more than five medications are considered the limitations of the study.

## Financial support

This work was funded by grant from National Natural Science Foundation of China (No. 81603200).

## Author's contribution

Jun Zhao, Lei Hu, Lihua Zhang, Maosong Zhou, and Lichen Gao: obtaining, analyzing and interpreting the data; effective participation in research orientation; intellectual participation in propaedeutic and therapeutic conduct of the cases studied; critical review of the literature.

Lin Cheng: conception and planning of the study; elaboration and writing of the manuscript; obtaining, analyzing and interpreting the data; effective participation in research orientation; intellectual participation in propaedeutic and therapeutic conduct of the cases studied; critical review and final approval of the manuscript.

## Conflicts of interest

None declared.
